# Evaluation of Different Formulations on the Viability of Phages for Use in Agriculture

**DOI:** 10.3390/v16091430

**Published:** 2024-09-07

**Authors:** Marcela León, Jorge Araya, Mauricio Nuñez, Manuel Arce, Fanny Guzmán, Carolina Yáñez, Ximena Besoain, Roberto Bastías

**Affiliations:** 1 Instituto de Biología, Facultad de Ciencias, Pontificia Universidad Católica de Valparaíso, Valparaíso 2340025, Chile; marcela.leon@pucv.cl (M.L.); mauricio.nunez@pucv.cl (M.N.);; 2 Núcleo de Biotecnología de Curauma, Pontificia Universidad Católica de Valparaíso, Valparaíso 2340025, Chile; 3 Laboratorio de Fitopatología, Escuela de Agronomía, Pontificia Universidad Católica de Valparaíso, Valparaíso 2340025, Chile

**Keywords:** *Pseudomonas syringae* pv. *actinidiae*, lyophilization, Psa, biological control, phage formulation

## Abstract

Bacteriophages have been proposed as biological controllers to protect plants against different bacterial pathogens. In this scenario, one of the main challenges is the low viability of phages in plants and under adverse environmental conditions. This work explores the use of 12 compounds and 14 different formulations to increase the viability of a phage mixture that demonstrated biocontrol capacity against *Pseudomonas syringae* pv. *actinidiae* (Psa) in kiwi plants. The results showed that the viability of the phage mixture decreases at 44 °C, at a pH lower than 4, and under UV radiation. However, using excipients such as skim milk, casein, and glutamic acid can prevent the viability loss of the phages under these conditions. Likewise, it was demonstrated that the use of these compounds prolongs the presence of phages in kiwi plants from 48 h to at least 96 h. In addition, it was observed that phages remained stable for seven weeks when stored in powder with skim milk, casein, or sucrose after lyophilization and at 4 °C. Finally, the phages with glutamic acid, sucrose, or skim milk maintained their antimicrobial activity against Psa on kiwi leaves and persisted within kiwi plants when added through roots. This study contributes to overcoming the challenges associated with the use of phages as biological controllers in agriculture.

## 1. Introduction

Lytic bacteriophages, as natural predators of bacteria, have been proposed as natural antimicrobials to combat pathogenic bacteria in various contexts, including their use in human health [1], food safety [2], aquaculture [3], and agriculture [4]. In all these cases, there are numerous experimental examples demonstrating their effectiveness [5,6,7,8,9], but at the same time, highlighting the challenges associated with the use of this technology in each area [10,11,12,13].

Particularly in agriculture, there are reports on the experimental use of phages to combat infections caused by various phytopathogenic bacteria such as *Ralstonia solanacearum* [14,15,16,17], *Xanthomonas arboricola* pv. *juglandis* [18,19], *Xanthomonas oryzae* pv. *oryzae* [20], *Agrobacterium tumefaciens* [21], and also against different pathovars of *Pseudomonas syringae*, such as *Pseudomonas syringae* pv. *syringae, Pseudomonas syringae* pv. *morsprunorum* [22], *Pseudomonas syringae* pv. *porri* [23], *Pseudomonas syringae* pv. *tomato*, and also *Pseudomonas syringae* pv. *actinidiae* that infects kiwi plants [24]. In most cases, one of the major challenges has been the short viability of phages under harsh environmental conditions [10,25,26,27,28,29]. In the agricultural context, phages (and other biocontrollers) can be exposed for long periods to solar radiation and other environmental factors that may inactivate them [25,30,31].

Recent studies show that phages, like Medea1, offer a viable and environmentally friendly alternative to chemical pesticides, significantly reducing bacterial speck symptoms in tomatoes through root drenching and foliar spraying. This approach also activates plant defense responses, but phage persistence remains challenging, as phages without excipients often fail to maintain long-term efficacy in field conditions [32].

Numerous reports addressed the use of different types of compounds to increase the viability of phages against adverse conditions [26,33,34] or improve their viability during storage [33,35]. One of the most extensively evaluated compounds with positive results is skim milk [30,34,36,37], but others, such as sucrose [30,38], glutamic acid [39,40] and trehalose [14,41], have also been evaluated. However, it has also been observed that not all phages respond similarly to the same compound [29,30,42], so it is necessary to evaluate different formulations for each case.

Recently, our team reported the use of bacteriophages as biocontrollers against *Pseudomonas syringae* pv. *actinidiae*. The effectiveness of the phages was evaluated both in vitro and in vivo in a greenhouse [24]. It was found that the phages used were rapidly inactivated in the plant, as reported in other studies [30], and they could not be detected after 48 h. This background motivated us to evaluate the use of different compounds that could increase the viability of phages under different environmental conditions.

This study evaluates the use of different formulations to increase the viability of *Pseudomonas syringae* pv. *actinidiae* phages in vitro and in kiwi plants under adverse environmental field conditions. Finally, the effect of different storage conditions was assessed on the viability of this phage mixture. This study represents a step forward in positioning phages as a viable and effective alternative for controlling phytopathogenic bacteria in agriculture.

## 2. Materials and Methods

### 2.1. Bacteria, Phages and Cultures

The bacterial strain *Pseudomonas syringae* pv. *actinidiae* 889 (Psa 889) used in this study has been previously characterized [43]. The phages CHF1, CHF7, CHF19, and CHF21 used in this study were also previously characterized [24]. All the assays were performed with a mixture of the different phages in equal proportion. The bacteria were grown in an LB medium (0.5% NaCl, 1% tryptone, and 0.5% yeast extract); the solid medium was supplemented with 1.5% agar. The phages were propagated as described previously [24], and the concentration of phages was determined by the double-agar assay [44]. Briefly, 100 μL of an early exponential bacterial culture was added to 3 mL of soft agar (LB, 0.6% agar). After gentle mixing, the culture was poured onto LB-agar plates to form a bacterial top-agar layer, left to solidify at room temperature, and incubated at 25 °C for 24 h.

### 2.2. Viability of Phages in Different Excipients

Based on a bibliographic search and their availability, 12 compounds were selected to evaluate their protective effect on phages because they have been used in other studies as excipients with phages, proteins, or other types of controllers. The list of the different excipients evaluated with the corresponding concentration and reference is presented in Table 1. The impact of these compounds on the viability of a phage mixture was evaluated using 10 µL of mix (with an initial concentration of 1 × 10^9^ PFU/mL) diluted in 990 µL of each excipient. The mixture was incubated at 25 °C for 24 h. After this period, the double-layer agar technique was used to determine the phage concentration of each case. All assays were performed in triplicates, and phages diluted in distilled water were used as a control.

### 2.3. Viability of Phages under Different Environmental Conditions and Protective Effect of Different Excipients

Three conditions—temperature, pH, and UV radiation—were evaluated to determine the viability of phages under different environmental conditions and the protective effect of the different excipients. All assays were conducted in triplicate unless otherwise stated.

Temperature: For the temperature assays, phages diluted in the corresponding excipient solution (~1 × 10^7^ PFU/mL) were incubated for 1 h at the corresponding temperatures. After this period, the phage titers were assessed using the double-layer agar technique.

pH: For the pH assays, 100 µL of phages diluted in the corresponding excipient solution (~1 × 10^7^ PFU/mL) were combined with 900 µL of sterile distilled water adjusted to the corresponding pH. The mixtures were incubated at 25 °C and the corresponding pH for 1 h. Subsequently, each mix was titrated using the double-layer agar spot technique.

UV Radiation: To evaluate the effect of UV radiation, phages diluted in the corresponding excipient solution (~1 × 10^7^ PFU/mL) were placed into the wells of a 12-well plate and exposed to UVC radiation for 0, 15, 30, and 60 min. (1 min. is equivalent to 120 J*m^−2^) in a laminar flow hood biosafety level 2 (BIOBASE Model 11231BBC86 MSC Class II) with a germicidal lamp that has a flux of 2.0 J*m^−2^S^−1^. Afterward, the phage titers for each time condition were determined using the double-layer agar spot technique.

### 2.4. Viability of Phages on Kiwi Plants under Field Conditions

These assays were conducted on kiwi vineyards (*Actinidia chinensis* var. *deliciosa* “Hayward”) located at the Experimental Station La Palma, which belongs to the Escuela de Agronomía, Pontificia Universidad Católica de Valparaíso, La Palma, Quillota, Chile. Four plants per treatment and five branches per plant were randomly selected and marked for phage application. A total of 1 L of phages (~1 × 10^7^ PFU/mL) diluted in the corresponding excipients was manually sprayed in the corresponding branches, using approximately 30 mL per branch (seven leaves per branch). The experiments were conducted for two consecutive weeks to evaluate the different formulations. In the first week, 5% glutamic acid, 0.1 M trehalose, 5% skim milk, and a mixture of them were evaluated. In the second week, 2% hydrolyzed casein, 0.5 M sucrose, 0.5% gelatin, and 0.5 M mannitol were evaluated. Each time, a control was included with phages diluted in water. These trials were conducted in January during the summer in the southern hemisphere.

To evaluate the presence of phages in the treated kiwi plants, five leaves were collected from each marked branch one hour post-application and every 24 h for five days (96 h). The five leaves from each plant were placed in a zip-lock plastic bag, weighed, and 10 mL of 10 mM MgSO_4_ buffer was added to each bag. The leaves were carefully washed with the buffer, and 5 mL from each bag was collected and filtered through 0.22 µm filters. Then, phage concentration was determined using the double-layer agar spot technique. During the assays, several leaves were collected from plants that did not receive treatment to discard the presence of native phages on those kiwi plants.

### 2.5. Evaluation of Different Storage Conditions

The viability of phages diluted in the different excipient solutions (1 × 10^9^ PFU/mL) under different storage conditions was evaluated in both solid and liquid conditions. The solid matrix of phages (powder) was obtained by lyophilization at the Curauma Biotechnology Center (NBC) of the Pontificia Universidad Católica de Valparaíso. Briefly, 1 mL of the corresponding formulation was added to 1.5 mL tubes and stabilized for 1 h at 4 °C. Subsequently, the samples were treated with liquid nitrogen and incubated for 1 h at −80 °C. Later, the first lyophilization cycle was carried out at −20 °C for 24 h in a pressure chamber of 100 mTorr. Then, the second cycle continued, maintaining the vacuum while the temperature was gradually increased over 10 h to 25 °C. Afterward, the samples were kept at 25 °C for 6 h until the pressure stabilized [48]. Immediately after lyophilization, the titer of the phages in the resulting solid matrices was determined and compared with the untreated phages. Both liquid (untreated) and solid formulations of phages were stored at 4 °C and 25 °C to monitor their viability over 7 weeks. Measurements were taken at weeks 0, 1, and then every 2 weeks until the end of the experiment. All assays were performed in triplicate.

### 2.6. Effectiveness of Phages with Different Excipients in Kiwifruit Leaf Discs

The efficacy of a phage mixture in various excipients against Psa 889 was evaluated using a leaf disc assay with modifications [24,49]. The discs were obtained from healthy kiwi plant (*Actinidia deliciosa* var. Hayward) leaves. Four excipients were selected for this assay: 5% glutamic acid, 5% skim milk, 0.5 M sucrose, and water. Each excipient contained the phage mixture at a titer of 1 × 10⁹ PFU/mL.

Leaves disinfection: Each leaf was disinfected with 75% ethanol for 3 min, followed by immersion in 1% sodium hypochlorite for 5 min. Subsequently, the leaves were washed five times with sterile distilled water and then once with a solution of cycloheximide (50 mg/L) for 3 min. After washing, the leaves were dried in a biosafety cabinet before cutting them into 1 × 1 cm discs. The discs were exposed to UV-C radiation for 5 min on each side. They were then placed individually in the wells of 6-well plates containing 2 mL of a sterile distilled water solution supplemented with cycloheximide (50 mg/L). Despite the disinfection, bacteria with different colony morphology were detected in the assay; the Psa colonies were confirmed by their morphology and by specific PCR [50].

Discs Assay: For each disc, a triplicate of Psa 889 was applied at a final concentration of 1 × 10⁷ CFU/mL. Each excipient was then separately applied, adjusting the titer to 1 × 10⁸ PFU/mL (MOI = 10). The discs were incubated at 25 °C. Bacterial load and phage concentration were determined at 24- and 48-h post-treatment, using different sets of plates for each time point. The assay was performed in triplicates.

### 2.7. Artificial Inoculation of Phages and Detection inside of Kiwi Plants

This assay aimed to evaluate the ability of phages to enter plant tissue and determine if the excipients enhance their viability within the plant. For this purpose, 45-day-old kiwi seedlings (*Actinidia deliciosa* var. Hayward) were used. Four excipients were tested: 5% glutamic acid, 5% skim milk, 0.5 M sucrose, and water. Each excipient contained the phage mixture at a final total concentration of 1 × 10⁸ PFU/mL.

To detect the phages within the kiwi plants, we first inoculated the phages in the plant roots [51]. To facilitate phage entry into the plant, artificial wounds were made in the main roots. Subsequently, 30 mL of each phage formulation was added directly to the soil around the roots, and the process was repeated every 12 h. The plants were maintained in a growth chamber with a 16 h light and 8 h dark cycle and 63% relative humidity. The experimental design included 15 plants, with 3 plants per treatment. The presence of phages in kiwi plants was determined directly from leaves and with enrichment using the host bacteria. Briefly, leaves were collected at 6, 24, and 48 h and then cut and incubated in 2 mL of 10 mM MgSO_4_ buffer at room temperature for 30 min. Then, the presence of phages was evaluated in the buffer using the double-layer agar assay, presenting the results as PFU/g of the leaf. For enrichment, 30 mL of fresh LB medium and 1 mL of Psa 889 (1 × 10⁸ CFU/mL) were added to the leaf samples. The enrichment was incubated for 12 h and then centrifuged and filtered for phage detection using the double-layer agar assay. The results were presented as the presence or absence of phages in the corresponding plant. Three plants were evaluated for each formulation.

### 2.8. Statistical Analysis

Statistical significance was determined using a two-way analysis of variance (ANOVA) (*p* < 0.05) with GraphPad Prism V.10.2.3. Only statistically significant differences are noted in the different figures. All experiments were performed in triplicate unless otherwise stated.

## 3. Results

### 3.1. Viability of Phages under Different Environmental Conditions

In a previous study, our team reported the effectiveness of a mixture of phages (CHF1, CHF7, CHF19, and CHF21) in combating infections caused by *Pseudomonas syringae* pv. *actinidiae* [24]. In the same study, it was observed that the phage mixture had very low viability on kiwi plants (48 h or less). Considering this background, our first approach consisted of evaluating the effect of environmental conditions on the viability of the phage mixture.

**Figure 1:**
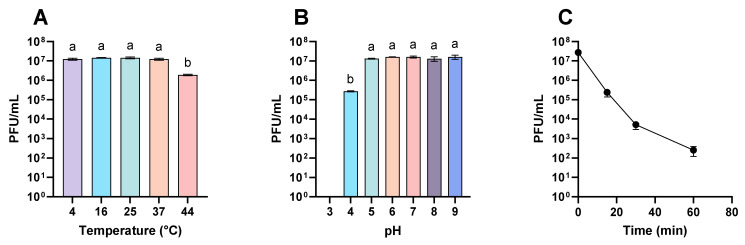
Viability of phages at different temperatures, pH, and UV radiation. The phage mixture in distilled water was incubated under different environmental conditions. (**A**) The concentration of phages after 1 h incubation in distilled water at the corresponding temperature. (**B**) The concentration of phages after 1 h incubation in distilled water was adjusted at the corresponding pH at 25 °C. (**C**) The concentration of phages after incubation in distilled water under UV radiation in a laminar flow hood for 0, 15, 30, or 60 min at 25 °C. The experiments were performed in triplicate, and the standard deviation bars are shown. Statistical analysis was performed using a 2-way ANOVA. The different letters in the bars indicate statistical differences (*p* < 0.0001).

The results showed that the phages remained stable within a temperature range of 4 °C to 37 °C for one hour. However, at 44 °C, their viability decreased by more than 6 times (Figure 1A). Higher temperatures were not evaluated, as it would be highly unlikely for phages to be exposed to temperatures exceeding 44 °C within a kiwi cultivation context. On the other hand, when evaluating the effect of pH, it was observed that the phage mixture remained stable in a pH range from 5 to 9, indicating resistance to basic conditions (Figure 1B). Conversely, the phage titer was reduced nearly 50 times at pH 4, and at pH 3, they were not detected (detection limit = 100 PFU/mL) (Figure 1B), suggesting that acidic conditions significantly affect the viability of the phage mixture.

Finally, the stability of the phages under UV radiation was also evaluated. After one hour, the titer of the mixture decreased rapidly from 2.7 × 10^7^ PFU/mL to 2.5 × 10^2^ (Figure 1C). These results indicate that the viability of the phage mixture is primarily affected by high temperatures, acidic conditions, and exposure to UV radiation.

### 3.2. Viability of Phages Suspended with Different Excipients under Different Environmental Conditions

To enhance the resistance of the phage mixture against the evaluated environmental conditions, a bibliographic search was conducted for various compounds acting as excipients that could increase phage viability. From this search, 14 formulations (12 excipients) were selected for evaluation (Table 1). Initially, the effect of these compounds on the phage mixture viability was assessed, revealing that some of them alone could decrease phage viability (Figure S1). For instance, it was observed that the phage mixture dissolved in 0.5 M PVP or 1% PABA was completely inactivated, while compounds such as EDTA (1 mM and 10 mM), PEG 6000 (5%), or glycerol (30% and 50%) also reduced the concentration of the phage mixture by 1 to 4 orders of magnitude (Figure S1). This analysis enabled the selection of the different formulations for the following assays, which included 0.5% gelatine, 0.5 M sucrose, 0.5 M mannitol, 2% casein, 5% skim milk, 0.1 M trehalose, and 5% glutamic acid. Only the environmental conditions that negatively affected the viability of phages were used to evaluate the protective effect of the different selected compounds.

**Figure 2:**
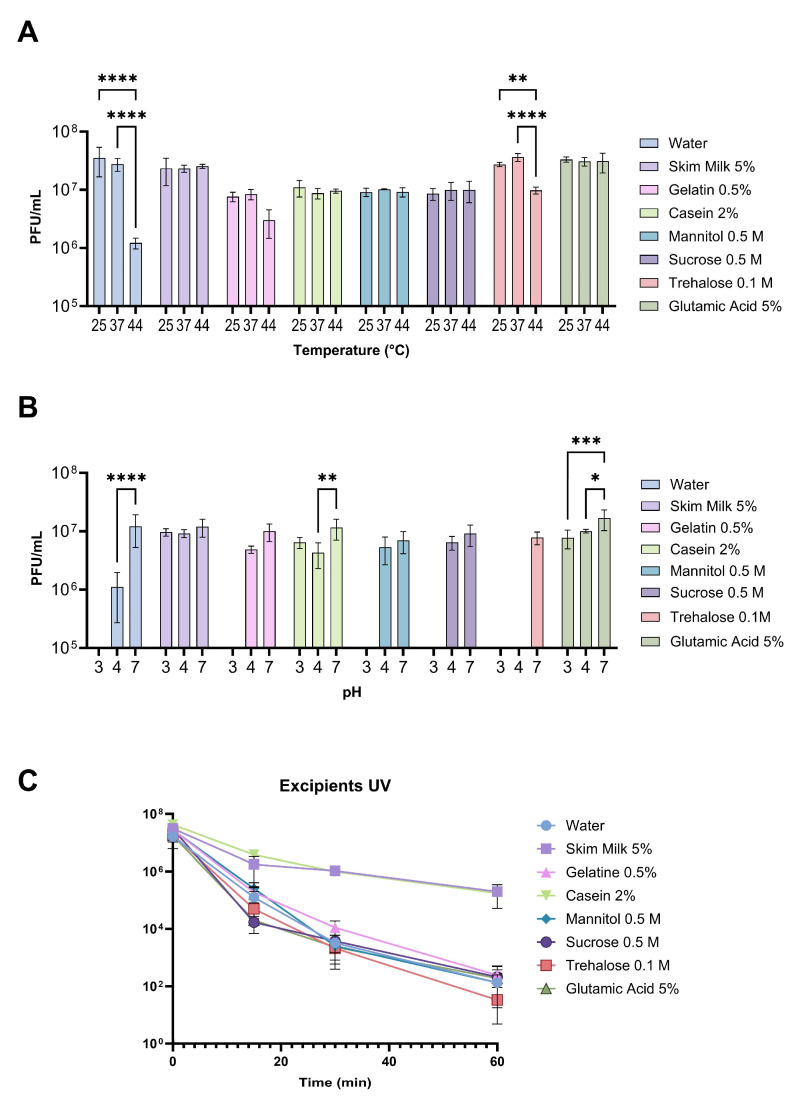
Viability of phages in different excipients under different environmental conditions. The mix of phages in the different excipients was incubated under different environmental conditions of temperature (**A**), pH (**B**), and exposition to UV radiation (**C**). The experiments were performed in triplicate, and the standard deviation is shown. The detection limit of the assay was 100 PFU/mL. Error bars represent the standard deviation. Statistical analysis was performed using a 2-way ANOVA. Symbols denote significant differences (*: *p* < 0.05; **: *p* < 0.01; ***: *p* < 0.001; ****: *p* < 0.0001).

When assessing the protective effect of the different selected compounds on phage viability exposed to various temperatures for one hour, it was observed that 5% skim milk, 2% casein, 0.5 M mannitol, 0.5 M sucrose, and 5% glutamic acid were able to maintain phage viability at 44 °C (Figure 2A). However, with 0.1 M trehalose at that temperature, a 2.79-fold reduction in phage concentration was observed compared to the concentration at 25 °C. With 0.5% gelatin, a 2.5-fold reduction in the concentration of the phage mixture at 44 °C was also observed, although this difference was not statistically significant.

Regarding pH, only 5% skim milk, 2% casein, and 5% glutamic acid exhibited a protective effect on phages at pH 3, although in the latter case, a 2.1-fold reduction was observed compared to the phage concentration observed at pH 7 (Figure 2B). All the compounds showed a protective effect at pH 4, although in some cases, a reduction in concentration was also observed compared to what was observed at neutral pH. In the case of 0.1 M trehalose, no phages were detected at pH 4 or 3 (detection limit 100 PFU/mL), indicating poorer performance than observed with phages resuspended only in water (Figure 2B).

Finally, when evaluating the protective effect of the different compounds when exposing the phage mixture to UV radiation, it was observed that only 2% casein and 5% skim milk presented a protective effect compared to phage viability when dissolved in water. After 60 min of exposure to UV radiation, phages with 2% casein and 5% skim milk maintained a phage concentration in the order of 10^5^ PFU/mL, while phages dissolved in water only maintained a concentration in the order of 10^2^, resulting in a reduction of 5 orders of magnitude in that period (Figure 2C). However, no differences were observed between the rest of the compounds and the water control. These results showed that some compounds, such as 5% skim milk, 2% casein, and, in some cases, 5% glutamic acid, can protect the phage mixture against adverse environmental conditions.

### 3.3. Viability of Phages on Kiwi Plant Surface under Field Conditions

**Figure 3:**
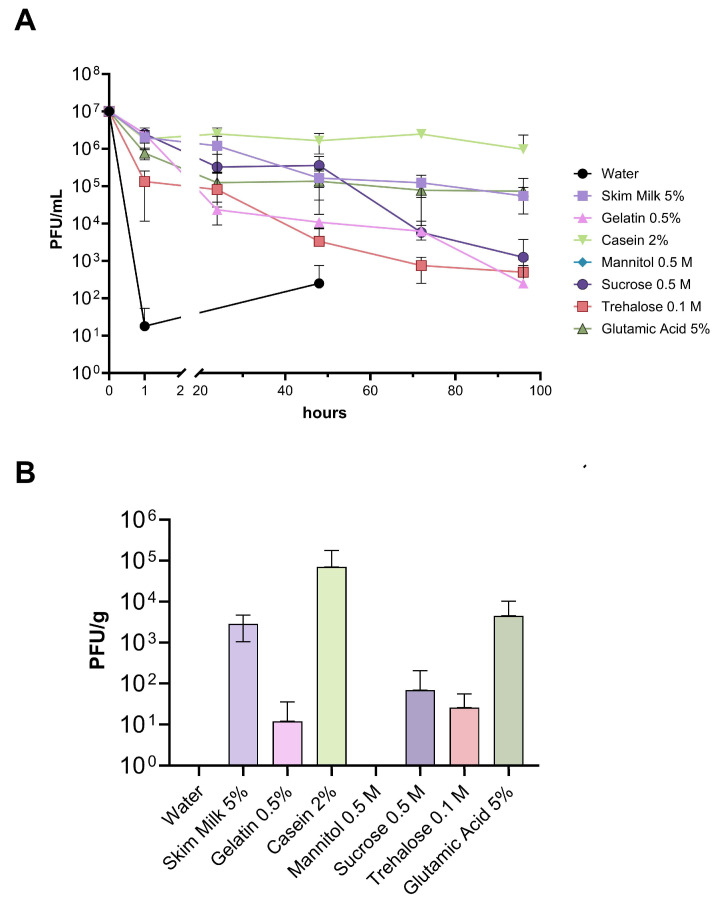
Viability of phages with the different excipients in kiwi plants. (**A**) The concentration of phages with the different excipients recovered from kiwi leaves at the corresponding time. Samples were collected throughout the experiment, and the points where no phage was detected were not plotted. The experiment detection point was 100 PFU/mL. (**B**) Concentration of phages per gram of leaf 96 h post-application with the different excipients. Phages were applied manually by aspersion with four replicates per treatment. Kiwi plants that did not receive treatment were used as controls to discard the presence of native phages. The error bars represent the standard deviation.

After evaluating the protective effect of the different compounds in vitro, the formulations were evaluated in vivo in kiwifruit orchards. In this case, the mixture of phages dissolved in water was only detected up to 48 h after inoculation and at a concentration near five orders of magnitude lower than the initial concentration of phages used (Figure 3A). On the other hand, among all the formulations evaluated, except for 0.5 M mannitol, phages were detected up to 96 h after starting the assay. The greatest protective effect was observed with 2% casein, followed by the formulation containing a mixture of 0.1 M trehalose + 5% glutamic acid + 5% skim milk. A protective effect was also observed with 5% skim milk alone or 5% glutamic acid alone, although it was less pronounced than in the previous cases. The lowest protective effect was observed with 0.5 M mannitol, where phages were not detected at any time during the experiment. The titer of phages with the formulations with 0.1 M trehalose alone or 0.5% gelatin decreased steadily until reaching the lowest levels at the end of the experiment (Figure 3A). The formulation with 0.5 M sucrose presented a protective effect comparable to that of skim milk and glutamic acid during the first 48 h of the assay but then decreased rapidly. Furthermore, it was observed in this case that the application of this compound attracted insects to the plants (Figure S2).

At the end of the trial, the highest concentrations of phages were found with the formulations of 2% casein, the mixture of trehalose + glutamic acid + sucrose, glutamic acid, and skim milk, with phage concentrations per gram of kiwi leaf of 7 × 10^4^ PFU/g, 1.2 × 10^4^ PFU/g, 4.5 × 10^3^ PFU/g, and 2.8 × 10^3^ PFU/g, respectively (Figure 3B). These results indicate that some of the evaluated formulations effectively allow the presence of the phage mixture to be extended on kiwi plants under real production conditions.

### 3.4. Evaluation of Different Storage Conditions

**Figure 4:**
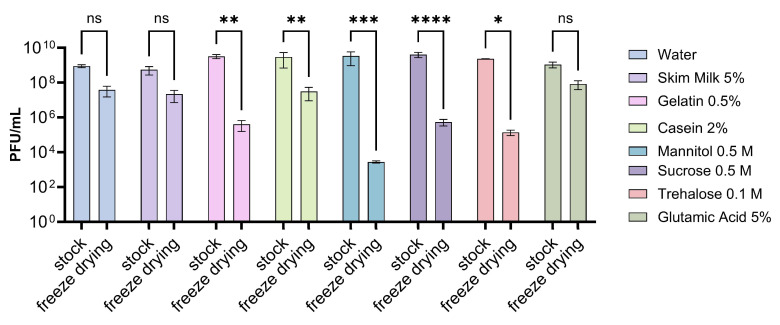
Impact of the lyophilization process on the viability of phages with the different excipients. The concentration of phages lyophilized and non-lyophilized with the different excipients was determined. The detection limit of the technique was 100 PFU/mL. The experiments were performed in triplicate, and the standard deviation is shown. Error bars represent the standard deviation. Statistical analysis was performed using a 2-way ANOVA. Symbols denote significant differences (ns: not significant; *: *p* < 0.05; **: *p* < 0.01; ***: *p* < 0.001; ****: *p* < 0.0001).

Finally, in this study, we aimed to evaluate different storage conditions for this phage mixture, considering the various formulations used. To achieve this, the different phage formulations were processed by a freeze-drying treatment, wherein we observed its impact on the viability of the phages. In all cases, a reduction in the concentration of phages was noted compared to the concentration before treatment (Figure 4). However, in the case of the control with water and in the formulations containing 5% skim milk or 5% glutamic acid, the decrease in the concentration of the phage mixture was not significant. On the other hand, a significant decrease in phage concentration was observed for 0.5% gelatin, 2% casein, 0.5 M sucrose, 0.1 M trehalose, and 0.5 M mannitol, with a reduction of 6 orders of magnitude in the last case.

After lyophilization, the stability of various phage formulations over time was evaluated by comparing storage in liquid or solid form as a powder at 4 °C and 25 °C. The results indicate that in the case of the water control, phages rapidly lost viability when incubated at 25 °C, becoming undetectable after one week when stored in powder form and after five weeks when stored in liquid form. When stored at 4 °C, phages were detected throughout the assay, but their viability decreased over time, with a more pronounced drop observed in phages stored in powder form.

Regarding the different formulations evaluated, the phages were detected throughout the entire experiment, except in the case of phages stored at 25 °C in powder with mannitol or sucrose that were not detected beyond the fifth week (Figure S3E,F). With the excipients casein, mannitol, and sucrose, a clear trend was observed, suggesting that powder storage would provide greater stability to the phage mixture throughout the experiment, with 4 °C being the optimal storage temperature (Figure S3D–F). Phages tended to be less stable in liquid, especially at 25 °C.

With other formulations, such as skim milk or glutamic acid, variability was observed in the stability of the phage mixture stored under different conditions throughout the experiment. However, at the end of the test, it was again observed that the phages maintained their viability to a greater extent when stored in powder at 4 °C (Figure S3B,H). Finally, when using trehalose, it was observed that in most of the points evaluated, greater viability of the phages stored in powder was observed compared to those stored in liquid, although high variability was also seen throughout the assay (Figure S3G), which was also observed when using gelatin as an excipient (Figure S3C).

**Figure 5:**
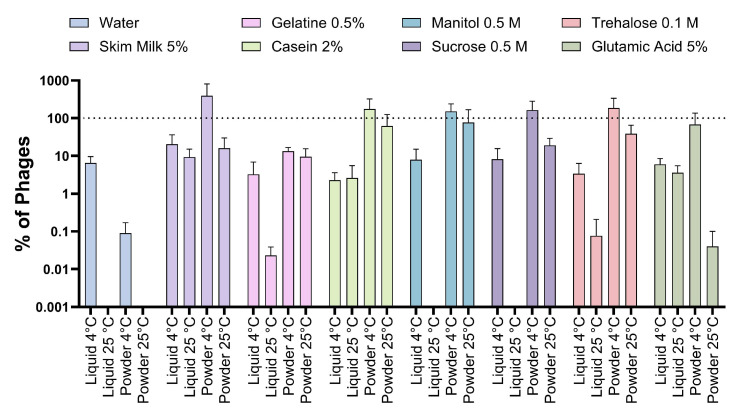
Viability of phages stored under different conditions after seven weeks. Phages were stored in liquid and solid conditions as a powder (lyophilized) at 4 °C and 25 °C for seven weeks with the different excipients evaluated. The titer of phages was normalized as a percentage of the initial titer. A segmented line represents 100%. Assays were performed in triplicate; error bars represent the standard deviation.

When analyzing the proportion of viable phages that persist at the end of the experiment in each condition, it was observed that in the majority of the formulations evaluated (six of seven), the viability of the phages remained viable in a proportion close to 100% when stored at 4 °C in powder (Figure 5), which contrasts with the control phage mixture whose highest viability occurred at 4 °C in liquid. On the other hand, the lower stability was mainly associated with the storage of phages in liquid at 25 °C. All these results showed that some of the excipients used provided greater stability to the phage mixture over time. Likewise, the results suggest that the best way to store phages together with excipients would be at 4 °C in powder form.

### 3.5. Effectiveness of Phages with Different Excipients to Control Psa and Persistence in Kiwi Plants

To evaluate whether the phages with the different excipients maintain their biocontrol activity against Psa 889, an in vitro assay was conducted using kiwifruit leaf discs. For this assay, skim milk, sucrose, and glutamic acid were selected as excipients based on their performance and the cost-effectiveness of a potential future industrial product. The results showed that at 24 h post-infection, only phages with 0.5 M sucrose reduced the load of Psa relative to the bacterial load in the control condition without phages. At 48 h post-infection, phages in water or with 5% skim milk or 5% glutamic acid reduced the Psa bacterial load by 79%, 95.7%, and 76.3%, respectively, relative to the control without phages (Figure S5). Despite the trend observed, no statistically significant differences were observed between the conditions, and a more robust analysis is needed to assess the effectiveness of the different formulations. In terms of phages, they were detected throughout the experiment in all conditions except the control without phages (Table S1).

**Figure 6:**
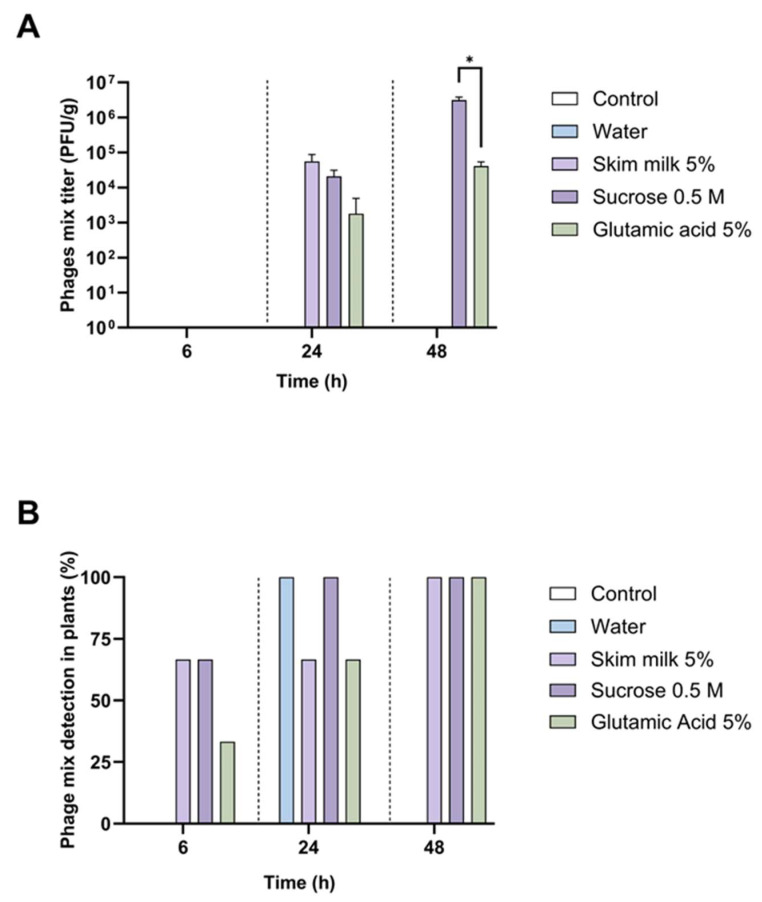
Persistence of phages with different excipients in kiwi plants. (**A**) Quantification of phages from kiwi plant leaves after inoculation in soil roots with the corresponding excipients. The detection limit of this assay was 200 PFU/g. (**B**) Detection of phages with enrichment with Psa 889 in kiwi plants after inoculation in soil roots. All experiments were performed in triplicate. Error bars represent the standard deviation. Statistical differences are presented (*: *p* < 0.05).

The persistence of the phages under endophytic conditions in kiwi plants was also evaluated. Following the initial inoculation of phages with the selected excipient in kiwi plant roots, the phages were detected and quantified in the leaves. At 6 h post-inoculation, no phages were detected, but at 24 h, 5.56 × 10^4^ PFU/g were detected with 5% skim milk, 2.08 × 10^4^ PFU/g with 0.5 M sucrose, and 1.79 × 10^3^ PFU/g with 5% glutamic acid, while at 48 h, phages were detected only with 0.5 M sucrose and 5% glutamic acid (3.15 × 10^6^ PFU/g and 4.10 × 10^4^ PFU/g, respectively) (Figure 6A). These results suggest that phages could spread within the kiwi plants when applied directly to the soil roots.

To evaluate whether phages were present under conditions where they cannot be quantified, that is, at concentrations below our detection limit (200 PFU/g), an enrichment approach with the host bacteria was used. In this case, at 6 h post-inoculation, phages with 5% skim milk or 0.5 M sucrose were present in 66% of plants tested and in just one plant when 5% glutamic acid was used as an excipient. At 24 h, phages were found in all plants with all the conditions evaluated, including phages with just water (Figure 6B). Finally, at 48 h, the phages were found in 100% of plants when combined with 5% skim milk, 0.5 M sucrose, or 5% glutamic acid. These results suggest that phages can spread through the kiwi plants, and the use of excipients can increase their persistence in the plant, as occurs on the leaf surface.

## 4. Discussion

This study shows the effect of different environmental conditions on a mixture of phages against *Pseudomonas syringae* pv. *actinidiae*. The results show that phages are sensitive to temperatures above 37 °C, pH below 5, and exposure to UV radiation. When evaluating different chemical compounds as excipients, it was observed that several of them can protect phages from these adverse environmental conditions, where casein, skim milk, and glutamic acid stand out. Likewise, it was shown that these formulations can help to extend the viability of phages on kiwi plants for up to 96 h. Finally, the best storage conditions for this phage mixture were also evaluated, determining that the conditions that provided greater stability over time corresponded to storage at 4 °C in powder form (Table S2).

Several studies have evaluated the use of different compounds to protect phages from adverse environmental conditions and during long storage periods. Compounds such as skim milk, sucrose, and casein, among others, have been studied with varying degrees of success [30,34,46,47,52]. This study evaluated 14 formulations (12 excipients), with disparate results being observed. Some compounds, such as skim milk, proved to be effective in increasing the viability of phages, which has also been observed in other studies [29,30,36]. On the contrary, compounds such as PVP reduced phage viability, which was also reported before [41]. On the other hand, excipients like sucrose showed good results in some of our assays, and other studies have shown no protective effect [48]. The discrepancy in relation to the protective effect of some compounds between different studies suggests that different phages do not respond in the same way to these treatments and excipients. In fact, Jo *et al*. [29] showed that compounds such as polysorbate 80 have different efficiencies protecting different phages from UV radiation [29], and similar results were observed by us in other studies [30,42]. This reinforces the need to carry out this type of study with phages or mixtures of phages that have potential as biocontrollers.

Several authors have reported that plants present an adverse environment for phages, and their viability is affected by numerous factors, such as pH, temperature fluctuations, and UV radiation [30,34]. Previous studies on this same phage mixture have reported their persistence in kiwi plants for up to 48 h [24]. Our findings revealed that phages indeed persist on kiwi plant leaves for only 48 h when applied with water alone; however, when different excipients were used, their presence on the plant surface extended for up to 96 h. Due to logistical constraints in accessing kiwi orchards, this trial could not be extended any further, so it cannot be conclusively stated if phages can persist in the plant for longer durations. Nevertheless, this persistence period in the plant would allow for weekly phage application schedules, aligning closely with the application schedules of other agricultural products.

It is widely known that phage viability can decrease over time, and that is why different storage methods have been evaluated. The storage of phages at −80 °C, with or without excipients, has proven to be very efficient for long periods [33], but this approach is impractical for application in products for agricultural use. On the other hand, numerous authors have shown that processes such as lyophilization can be useful to extend phage viability over time [33,35,53]. However, this procedure can be very aggressive and tends to reduce the concentration of the treated phages by around two orders of magnitude, but they are then able to remain stable for up to more than one year [14,41,45]. Likewise, different compounds have been evaluated, such as skim milk, gelatin, sucrose, or trehalose that help in protecting phages during the lyophilization process [35,41,42,45]. We verified that the freeze-drying process effectively reduced phage viability by at least one order of magnitude. In the present case, the best results were observed with skim milk and glutamic acid, although those results were not comparatively better than those obtained with the water control. Interestingly, some compounds, such as mannitol and trehalose, resulted in an even greater reduction of up to six orders of magnitude in the concentration of the phage mixture (Figure 4). In the case of trehalose, it was also observed that phages showed variation in their viability during storage at different conditions (Figure S3G). It has been reported that the lyophilized powder with trehalose tends to form crystals that affect the stability of the phages [54,55]. This could explain the poor results obtained with this compound, and something similar could occur with other excipients.

According to our results, the optimal condition for storing phages would be at 4 °C in lyophilized powder format, using skim milk, casein, mannitol, sucrose, or even glutamic acid as excipients. However, it is important to consider the loss of viability that phages undergo during the freeze-drying process. Since the viability analysis of the phage mixture over time was conducted immediately after the freeze-drying process, the final concentrations of the phage mixtures stored under each condition can be compared, taking into account the reduction in phage concentration due to freeze-drying (Figure S4). Notably, formulations containing skim milk, casein, or glutamic acid showed compensatory effects, where the loss in phage viability resulting from lyophilization was offset by the greater stability achieved by storing the phages in powder format with the excipient. Therefore, utilizing phage stocks with higher concentrations could lead to powder formulations with increased stability over time.

Our results showed that phages with 5% glutamic acid or 5% skim milk maintain antimicrobial activity against Psa in kiwi leaves; however, a more robust analysis is needed to assess the effectiveness of the different formulations (Figure S5). In this regard, it has been described that disaccharides like sucrose and amorphous polymers found in milk increase the structural viability of phages by replacing and stabilizing their interaction with the surrounding water, which translates to increased phage longevity [45,53,56,57]. However, excipients such as casein and skim milk, while potentially stabilizing for certain bacteriophages, might negatively affect the phage’s ability to infect its host [58,59]. On the other hand, some excipients, such as skim milk, sucrose, or even glutamic acid, may serve as energy and carbon sources for Psa [43], contrasting with the biocontrol activity of phages. This complex scenario of interactions could explain the high variability observed in the assays with leaf discs and advocates for further analysis that conducts a more precise formulation of phages. Nonetheless, the findings of this study suggest that excipients are crucial for phages to endure harsh environmental conditions.

The detection of phages in kiwi plants after inoculation in soil roots strongly suggests that phages can be transported from the root to aerial parts of the plant through the vascular system, as has been described by other authors [51,60,61]. This is further supported by findings that phage application through root drenching significantly reduced 2.5-fold Pseudomonas syringae pv. tomato symptoms in tomato plants, although foliar spraying proved to be more effective. This reinforces the idea that administering phages through irrigation systems can be a good alternative for agriculture [32]. Our results also suggest that excipients increase the persistence of phages inside the plants, offering probably systemic protection against pathogens. However, we have not evaluated biocontrol activity in these assays since plants were not infected with Psa. Therefore, further studies should focus on addressing the biocontrol activity of formulations with selected excipients and evaluating the systemic protection that these phages can offer [51,61].

This study contributes to the development of new formulations based on bacteriophages for use in agriculture. Further studies are needed to evaluate the effectiveness of selected formulations under field conditions and to evaluate different storage processes, such as spray drying [33,35,42].

## 5. Conclusions

The results presented in this study demonstrate that the use of different compounds can enhance the viability of a phage mixture against *Pseudomonas syringae* pv. *actinidiae* under adverse environmental conditions, as well as on kiwi plants. However, while the freeze-drying process may impact the viability of the phages, the incorporation of skim milk, casein, or glutamic acid contributes to their stability over time when stored in powder form at 4 °C. On the other hand, the phages with compounds such as skim milk, sucrose, or glutamic acid maintain antimicrobial activity against Psa on kiwi leaves and persist within kiwi plants after addition through soil root. Considering all the results obtained, it is estimated that a formulation containing skim milk, casein, sucrose, or glutamic acid would be most appropriate for composing a formulation with these phages.

## Figures and Tables

**Table 1:** List of excipients selected to evaluate their protective effect on phages under adverse environmental conditions.

Excipient	Concentration	Reference
Polyethylene glycol (PEG 600)	5% (*w*/*v*)	[41,45]
Glutamic acid	5% (*w*/*v*)	[39]
Skim milk	5% (*w*/*v*)	[42]
Casein	2% (*w*/*v*)	[23]
Gelatin	0.5% (*w*/*v*)	[39,45]
para-aminobenzoic acid (PABA)	0.1% (*w*/*v*)	[31]
Glycerol	50–30% (*w*/*v*)	[46]
Polyvinylpyrrolidone (PVP)	0.5 M	[41]
Ethylenediaminetetraacetic acid (EDTA)	1–10 mM	[47]
Mannitol	0.5 M	[41,45]
Sucrose	0.1 M	[41,42,45]
Trehalose	0.1 M	[41]

## Data Availability

All the data generated are available at the following link: https://doi.org/10.6084/m9.figshare.25859956.

## Supplementary Materials

The following supporting information can be downloaded at: https://www.mdpi.com/article/10.3390/v16091430/s1, Figure S1. Effect of different excipients on the viability of phages; Figure S2. Application of phages with 0.5 M sucrose to kiwi plants; Figure S3. Viability of phages stored under different conditions; Figure S4. Concentration of phages stored under different conditions after seven weeks; Figure S5. Effectiveness of phages with different excipients to control Psa; Table S1. Concentration of phages present in kiwi leaf discs assay; Table S2. Summary of results obtained in the different assays with the list of excipients evaluated in vitro and in vivo experiments. The data correspond to the average of the results obtained from the respective replicates.

## References

[1] Pirnay J.-P., Ferry T., Resch G. (2022). Recent Progress toward the Implementation of Phage Therapy in Western Medicine. FEMS Microbiol. Rev..

[2] Moye Z., Woolston J., Sulakvelidze A. (2018). Bacteriophage Applications for Food Production and Processing. Viruses.

[3] Nokhwal A., Anand T., Ravikant, Vaid R.K. (2023). Bacteriophage Therapy: An Emerging Paradigm in Fish Disease Management. Aquac. Int..

[4] Buttimer C., McAuliffe O., Ross R.P., Hill C., O’Mahony J., Coffey A. (2017). Bacteriophages and Bacterial Plant Diseases. Front. Microbiol..

[5] Svircev A., Roach D., Castle A. (2018). Framing the Future with Bacteriophages in Agriculture. Viruses.

[6] Sieiro C., Areal-Hermida L., Pichardo-Gallardo Á., Almuiña-González R., De Miguel T., Sánchez S., Sánchez-Pérez Á., Villa T.G. (2020). A Hundred Years of Bacteriophages: Can Phages Replace Antibiotics in Agriculture and Aquaculture?. Antibiotics.

[7] Green S.I., Clark J.R., Santos H.H., Weesner K.E., Salazar K.C., Aslam S., Campbell J.W., Doernberg S.B., Blodget E., Morris M.I. (2023). A Retrospective, Observational Study of 12 Cases of Expanded-Access Customized Phage Therapy: Production, Characteristics, and Clinical Outcomes. Clin. Infect. Dis..

[8] Bumunang E.W., Zaheer R., Niu D., Narvaez-Bravo C., Alexander T., McAllister T.A., Stanford K. (2023). Bacteriophages for the Targeted Control of Foodborne Pathogens. Foods.

[9] Córdova P., Rivera-González J.P., Rojas-Martínez V., Fiore N., Bastías R., Zamorano A., Vera F., Barrueto J., Díaz B., Ilabaca-Díaz C. (2023). Phytopathogenic *Pseudomonas Syringae* as a Threat to Agriculture: Perspectives of a Promising Biological Control Using Bacteriophages and Microorganisms. Horticulturae.

[10] Halawa E.M. (2023). Challenges of Bacteriophages Application in Controlling Bacterial Plant Diseases and How to Overcome Them. J. Genet. Eng. Biotechnol..

[11] Korniienko N., Kharina A., Budzanivska I., Burketová L., Kalachova T. (2022). Phages of Phytopathogenic Bacteria: High Potential, but Challenging Application. Plant Prot. Sci..

[12] Lomelí-Ortega C.O., Balcázar J.L., Quiroz-Guzmán E. (2023). Phage Therapy and Aquaculture: Progress and Challenges. Int. Microbiol..

[13] Fabijan A.P., Iredell J., Danis-Wlodarczyk K., Kebriaei R., Abedon S.T. (2023). Translating Phage Therapy into the Clinic: Recent Accomplishments but Continuing Challenges. PLoS Biol..

[14] Álvarez B., Gadea-Pallás L., Rodríguez A., Vicedo B., Figàs-Segura À., Biosca E.G. (2022). Viability, Stability and Biocontrol Activity *in Planta* of Specific *Ralstonia solanacearum* Bacteriophages after Their Conservation Prior to Commercialization and Use. Viruses.

[15] Umrao P.D., Kumar V., Kaistha S.D. (2021). Biocontrol Potential of Bacteriophage ɸsp1 against Bacterial Wilt-Causing *Ralstonia solanacearum* in *Solanaceae* Crops. Egypt. J. Biol. Pest Control..

[16] Huang B., Ge L., Xiang D., Tan G., Liu L., Yang L., Jing Y., Liu Q., Chen W., Li Y. (2024). Isolation, Characterization, and Genomic Analysis of a Lytic Bacteriophage, PQ43W, with the Potential of Controlling Bacterial Wilt. Front. Microbiol..

[17] Yang K., Wang X., Hou R., Lu C., Fan Z., Li J., Wang S., Xu Y., Shen Q., Friman V.-P. (2023). Rhizosphere Phage Communities Drive Soil Suppressiveness to Bacterial Wilt Disease. Microbiome.

[18] Retamales J., Núñez P., Alvarado R., Campan E.D.M., Otto T., Segovia C., Vasquez I., Santander J. (2022). Characterization of *Xanthomonas arboricola* Pv. *Juglandis* Bacteriophages against Bacterial Walnut Blight and Field Evaluation. Viruses.

[19] Kizheva Y., Urshev Z., Dimitrova M., Bogatzevska N., Moncheva P., Hristova P. (2023). Phenotypic and Genotypic Characterization of Newly Isolated *Xanthomonas euvesicatoria*-Specific Bacteriophages and Evaluation of Their Biocontrol Potential. Plants.

[20] Jain L., Kumar V., Jain S.K., Kaushal P., Ghosh P.K. (2023). Isolation of Bacteriophages Infecting *Xanthomonas Oryzae* Pv. *Oryzae* and Genomic Characterization of Novel Phage vB_XooS_NR08 for Biocontrol of Bacterial Leaf Blight of Rice. Front. Microbiol..

[21] Stonier T., McSharry J., Speitel T. (1967). *Agrobacterium tumefaciens* Conn. IV. Bacteriophage PB21 and Its Inhibitory Effect on Tumor Induction. J. Virol..

[22] Rabiey M., Roy S.R., Holtappels D., Franceschetti L., Quilty B.J., Creeth R., Sundin G.W., Wagemans J., Lavigne R., Jackson R.W. (2020). Phage Biocontrol to Combat *Pseudomonas syringae* Pathogens Causing Disease in Cherry. Microb. Biotechnol..

[23] Rombouts S., Volckaert A., Venneman S., Declercq B., Vandenheuvel D., Allonsius C.N., Van Malderghem C., Jang H.B., Briers Y., Noben J.P. (2016). Characterization of Novel Bacteriophages for Biocontrol of Bacterial Blight in Leek Caused by *Pseudomonas syringae* Pv. Porri. Front. Microbiol..

[24] Flores O., Retamales J., Núñez M., León M., Salinas P., Besoain X., Yañez C., Bastías R. (2020). Characterization of Bacteriophages against *Pseudomonas syringae* Pv. *Actinidiae* with Potential Use as Natural Antimicrobials in Kiwifruit Plants. Microorganisms.

[25] Jones J.B., Vallad G.E., Iriarte F.B., Obradović A., Wernsing M.H., Jackson L.E., Balogh B., Hong J.C., Momol M.T. (2012). Considerations for Using Bacteriophages for Plant Disease Control. Bacteriophage.

[26] Liu S., Quek S.-Y., Huang K. (2023). Advanced Strategies to Overcome the Challenges of Bacteriophage-Based Antimicrobial Treatments in Food and Agricultural Systems. Crit. Rev. Food Sci. Nutr..

[27] Frampton R.A., Pitman A.R., Fineran P.C. (2012). Advances in Bacteriophage-Mediated Control of Plant Pathogens. Int. J. Microbiol..

[28] Qin C., Tao J., Liu T., Liu Y., Xiao N., Li T., Gu Y., Yin H., Meng D. (2019). Responses of Phyllosphere Microbiota and Plant Health to Application of Two Different Biocontrol Agents. AMB Express.

[29] Jo S.J., Kim S.G., Park J., Lee Y.M., Giri S.S., Lee S.B., Jung W.J., Hwang M.H., Park J.H., Roh E. (2023). Optimizing the Formulation of *Erwinia* Bacteriophages for Improved UV Stability and Adsorption on Apple Leaves. Heliyon.

[30] Iriarte F.B., Balogh B., Momol M.T., Smith L.M., Wilson M., Jones J.B. (2007). Factors Affecting Survival of Bacteriophage on Tomato Leaf Surfaces. Appl. Environ. Microbiol..

[31] Hadapad A.B., Hire R.S., Vijayalakshmi N., Dongre T.K. (2009). UV Protectants for the Biopesticide Based on *Bacillus sphaericus* Neide and Their Role in Protecting the Binary Toxins from UV Radiation. J. Invertebr. Pathol..

[32] Skliros D., Papazoglou P., Gkizi D., Paraskevopoulou E., Katharios P., Goumas D.E., Tjamos S., Flemetakis E. (2023). In *Planta* Interactions of a Novel Bacteriophage against Pseudomonas syringae pv. tomato. Appl. Microbiol. Biotechnol..

[33] Malik D.J., Sokolov I.J., Vinner G.K., Mancuso F., Cinquerrui S., Vladisavljevic G.T., Clokie M.R.J., Garton N.J., Stapley A.G.F., Kirpichnikova A. (2017). Formulation, Stabilisation and Encapsulation of Bacteriophage for Phage Therapy. Adv. Colloid Interface Sci..

[34] Balogh B., Jones J., Iriarte F., Momol M. (2010). Phage Therapy for Plant Disease Control. CPB.

[35] Zhang Y., Zhang H., Ghosh D. (2020). The Stabilizing Excipients in Dry State Therapeutic Phage Formulations. AAPS PharmSciTech.

[36] Liu J., Wang H., Chia S.L., Tan G.H. (2022). Screening and Formulation of Novel Carriers for *Xanthomonas* Bacteriophage to Control Bacterial Leaf Blight Disease. MJM.

[37] Balogh B., Jones J.B., Momol M.T., Olson S.M., Obradovic A., King P., Jackson L.E. (2003). Improved Efficacy of Newly Formulated Bacteriophages for Management of Bacterial Spot on Tomato. Plant Dis..

[38] Gašić K., Kuzmanović N., Ivanović M., Prokić A., Šević M., Obradović A. (2018). Complete Genome of the *Xanthomonas euvesicatoria* Specific Bacteriophage KΦ1, Its Survival and Potential in Control of Pepper Bacterial Spot. Front. Microbiol..

[39] Engel H.W.B., Smith L., Berwald L.G. (1974). The Preservation of Mycobacteriophages by Means of Freeze Drying. Am. Rev. Respir. Dis..

[40] Davies J.D., Kelly M.J. (1969). The Preservation of Bacteriophage H1 of *Corynebacterium Ulcerans* U 103 by Freeze-Drying. Epidemiol. Infect..

[41] Merabishvili M., Vervaet C., Pirnay J.-P., Vos D.D., Verbeken G., Mast J., Chanishvili N., Vaneechoutte M. (2013). Stability of *Staphylococcus aureus* Phage ISP after Freeze-Drying (Lyophilization). PLoS ONE.

[42] González-Menéndez E., Fernández L., Gutiérrez D., Rodríguez A., Martínez B., García P. (2018). Comparative Analysis of Different Preservation Techniques for the Storage of *Staphylococcus* Phages Aimed for the Industrial Development of Phage-Based Antimicrobial Products. PLoS ONE.

[43] Flores O., Prince C., Nuñez M., Vallejos A., Mardones C., Yañez C., Besoain X., Bastías R. (2018). Genetic and Phenotypic Characterization of Indole-Producing Isolates of *Pseudomonas syringae* Pv. *Actinidiae* Obtained from Chilean Kiwifruit Orchards. Front. Microbiol..

[44] Kutter E., Clokie M.R.J., Kropinski A.M. (2009). Phage Host Range and Efficiency of Plating. Bacteriophages: Methods and Protocols, Volume 1: Isolation, Characterization, and Interactions.

[45] Manohar P., Ramesh N. (2019). Improved Lyophilization Conditions for Long-Term Storage of Bacteriophages. Sci. Rep..

[46] Clark W.A. (1962). Comparison of Several Methods for Preserving Bacteriophages. Appl. Microbiol..

[47] Brown T.L., Thomas T., Odgers J., Petrovski S., Spark M.J., Tucci J. (2017). Bacteriophage Formulated into a Range of Semisolid and Solid Dosage Forms Maintain Lytic Capacity against Isolated Cutaneous and Opportunistic Oral Bacteria. J. Pharm. Pharmacol..

[48] Puapermpoonsiri U., Ford S.J., van der Walle C.F. (2010). Stabilization of Bacteriophage during Freeze Drying. Int. J. Pharm..

[49] Prencipe S., Gullino M.L., Spadaro D. (2018). *Pseudomonas syringae* Pv. *Actinidiae* Isolated from *Actinidia chinensis* Var. Deliciosa in Northern Italy: Genetic Diversity and Virulence. Eur. J. Plant Pathol..

[50] Balestra G.M., Taratufolo M.C., Vinatzer B.A., Mazzaglia A. (2013). A Multiplex PCR Assay for Detection of *Pseudomonas syringae* Pv. *Actinidiae* and Differentiation of Populations with Different Geographic Origin. Plant Dis..

[51] Rahimi-Midani A., Choi T.-J. (2020). Transport of Phage in Melon Plants and Inhibition of Progression of Bacterial Fruit Blotch. Viruses.

[52] Orynbayev A.T., Dzhalilov F.S.U., Ignatov A.N. (2020). Improved Efficacy of Formulated Bacteriophage in Control of Black Rot Caused by *Xanthomonas campestris* Pv. *Campestris* on Cabbage Seedlings. Arch. Phytopathol. Plant Prot..

[53] Matinkhoo S., Lynch K.H., Dennis J.J., Finlay W.H., Vehring R. (2011). Spray-Dried Respirable Powders Containing Bacteriophages for the Treatment of Pulmonary Infections. J. Pharm. Sci..

[54] Vandenheuvel D., Meeus J., Lavigne R., Van den Mooter G. (2014). Instability of Bacteriophages in Spray-Dried Trehalose Powders Is Caused by Crystallization of the Matrix. Int. J. Pharm..

[55] Yan W., He R., Tang X., Tian B., Liu Y., Tong Y., To K.K.W., Leung S.S.Y. (2021). The Influence of Formulation Components and Environmental Humidity on Spray-Dried Phage Powders for Treatment of Respiratory Infections Caused by *Acinetobacter baumannii*. Pharmaceutics.

[56] Mehta B.M., Cheung P.C.K., Mehta B.M. (2015). Chemical Composition of Milk and Milk Products. Handbook of Food Chemistry.

[57] Wdowiak M., Paczesny J., Raza S. (2022). Enhancing the Stability of Bacteriophages Using Physical, Chemical, and Nano-Based Approaches: A Review. Pharmaceutics.

[58] Sitohy M., Chobert J.-M., Karwowska U., Gozdzicka-Jozefiak A., Haertlé T. (2006). Inhibition of Bacteriophage M13 Replication with Esterified Milk Proteins. J. Agric. Food Chem..

[59] García-Anaya M.C., Sepúlveda D.R., Rios-Velasco C., Zamudio-Flores P.B., Sáenz-Mendoza A.I., Acosta-Muñiz C.H. (2020). The Role of Food Compounds and Emerging Technologies on Phage Stability. Innov. Food Sci. Emerg. Technol..

[60] Ward R.L., Mahler R.J. (1982). Uptake of Bacteriophage F2 through Plant Roots. Appl. Environ. Microbiol..

[61] Iriarte F.B., Obradović A., Wernsing M.H., Jackson L.E., Balogh B., Hong J.A., Momol M.T., Jones J.B., Vallad G.E. (2012). Soil-Based Systemic Delivery and Phyllosphere in Vivo Propagation of Bacteriophages: Two Possible Strategies for Improving Bacteriophage Persistence for Plant Disease Control. Bacteriophage.

