# Supplementary material for: Evaluation of Different Formulations on the Viability of Phages for Use in Agriculture
Source: Viruses. 2024 Sep 7;16(9):1430. doi: 10.3390/v16091430 (PMC11437505; doi:10.3390/v16091430)
Supplement: Supplementary file 1 [file viruses-16-01430-s001.zip › viruses-3176390-supplementary.pdf]

Evaluation of different formulations on the viability of phages for use in agriculture.

Marcela León [1†], Jorge Araya[1†], Mauricio Nuñez [1] , Manuel Arce [1], Fanny Guzmán[2], Carolina Yáñez[1], Ximena Besoain[3] and Roberto Bastías [1,*]

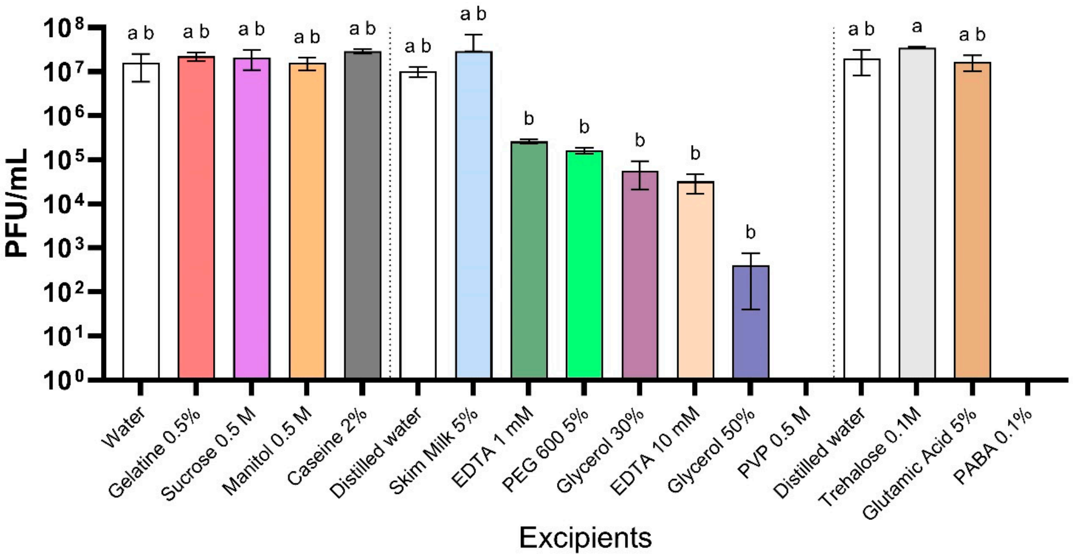

Figure S1. Effect of different excipients on the viability of phages. The graph shows the concentration of phages after 24 h of incubation in different excipients at 4 °C. The experiments were performed in triplicate, and standard deviation bars are shown. The detection limit of the experiment was 100 PFU/mL. Statistical analysis was performed using 2-way ANOVA. The different letters about the bars indicate statistical differences ($p < 0.05$).

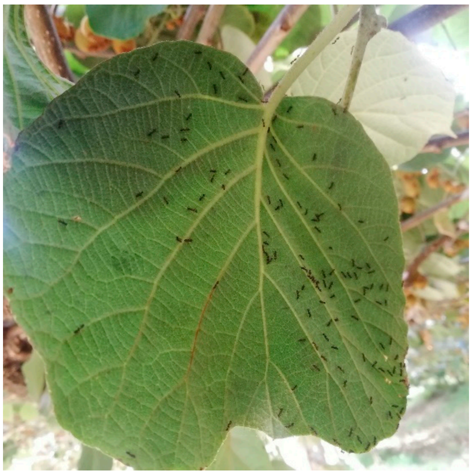

Figure S2. Application of phages with 0.5 M sucrose to kiwi plants. The image shows how the leaves that received this formulation were infested with insects one day post-application.

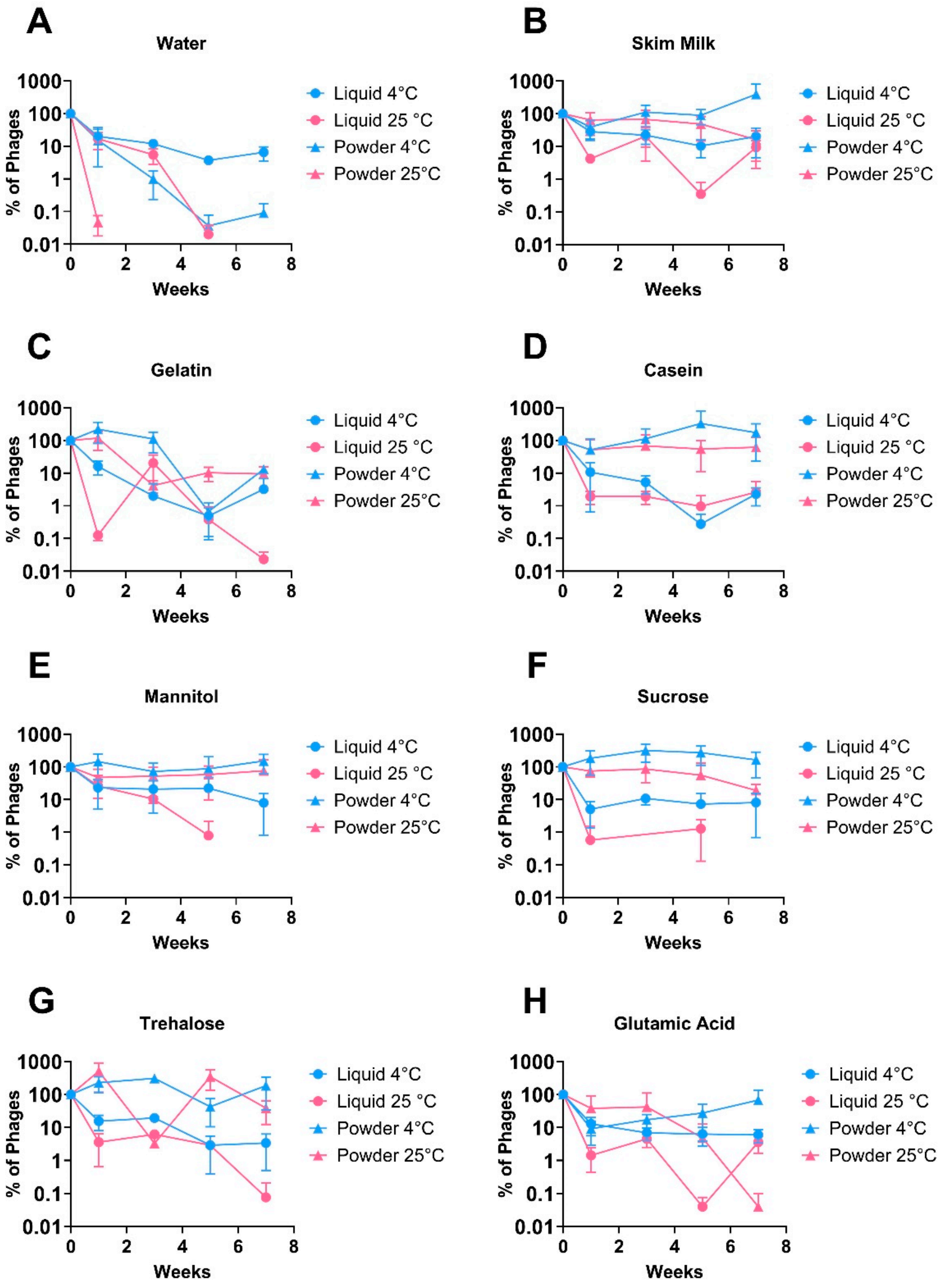

Figure S3. Viability of phages stored under different conditions. Phages were stored in liquid (circles) and solid condition as a powder (lyophilized) (triangles), at 4 °C (blue) and 25 °C (red) for seven weeks with the different excipients evaluated. Samples were collected throughout the experiment; points where phage were not detected were not plotted (detection limit 10 PFU/mL). The corresponding excipient used is shown on top of each graph. The phage concentration was

normalized as a percentage of the initial titer. Assays were performed in triplicate, error bars represent the standard deviation.

Table S1: Concentration of phages present in kiwi leaf discs assay.

Time (hour)	Distilled Water (PFU/mL)	5% Skim Milk (PFU/mL)	0.5 M Sucrose (PFU/mL)	5% Glutamic Acid (PFU/mL)
0	$6.00X10^{7}$	$9.33X10^{7}$	$6.00X10^{7}$	$3.33X10^{7}$
24	$2.73X10^{8}$	$2.22X10^{6}$	$3.23X10^{9}$	$1.23X10^{7}$
48	$3.42X10^{8}$	$6.00X10^{6}$	$2.17X10^{9}$	$1.25X10^{8}$

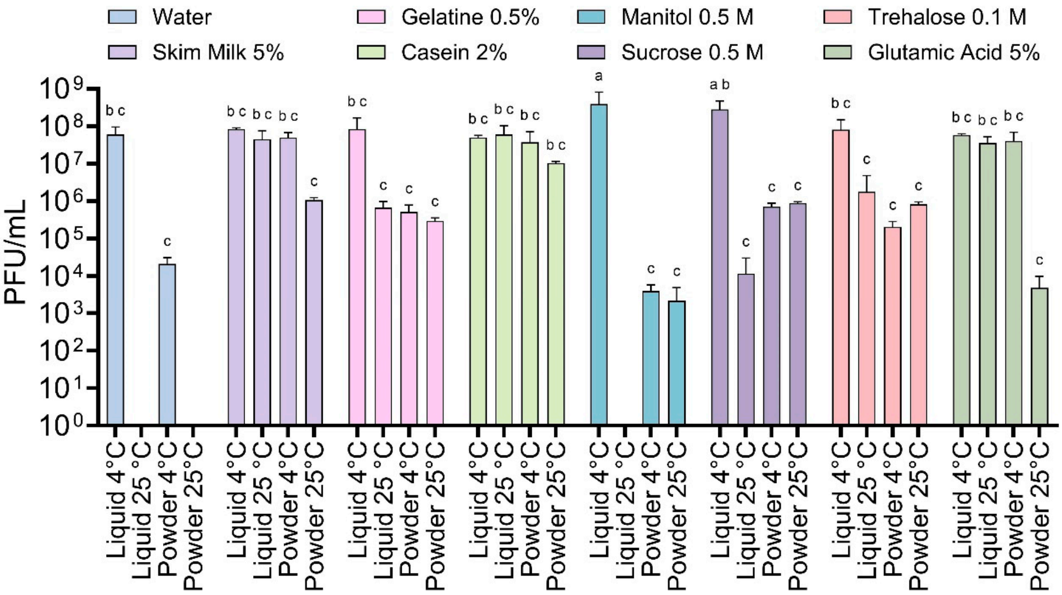

Figure S4. Concentration of phages stored under different conditions after seven weeks. Phages were stored in liquid and solid condition as a powder (lyophilized), at 4 °C and 25 °C for seven weeks with the different excipients evaluated. Assays were performed in triplicates; error bars represent the standard deviation. Statistical analysis was performed using 2-way ANOVA. The different letters about the bars indicate statistical differences ($p < 0.05$).

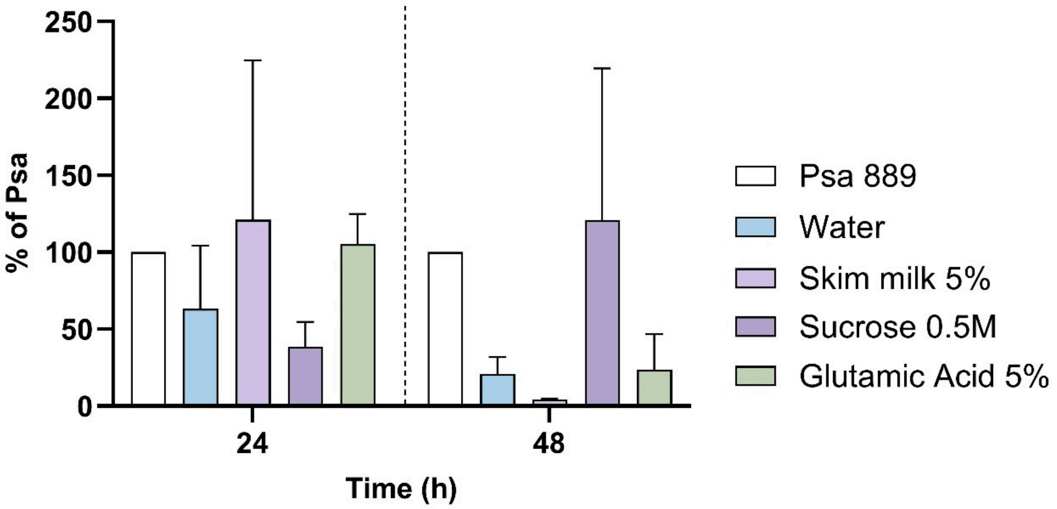

Figure S5. Effectiveness of phages with different excipients to control Psa. Percentage of Psa load in kiwi leaf disc inoculated with Psa and phages at MOI=10 with the corresponding excipients. The percentage of bacteria is presented relative to the bacterial load in the control without phages. All experiments were performed in triplicate. Error bars represent the standard deviation. Statistical differences are presented (*: $p<0.05$).

Table S2: Summary of results obtained in the different assays with the list of excipients evaluated *in vitro* and *in vivo* experiments. The data correspond to the average of the results obtained from the respective replicates

	Condition	Distilled Water	5% Skim Milk	0.5% Gelatin	2% Casein	0.5 M Mannitol	0.5 M Sucrose	0.1 M Trehalose	5% Glutamic Acid
pH[a]	pH 3	0	9.66×10^6	0	6.44×10^6	0	0	0	7.76×10^6
	pH 4	1.11×10^6	9.19×10^6	4.89×10^6	4.31×10^6	5.32×10^6	6.44×10^6	7.07×10^3	1.01×10^7
	pH 7	1.22×10^7	1.20×10^7	9.99×10^6	1.16×10^7	6.99×10^6	9.10×10^6	7.78×10^6	1.68×10^7
Temperature[a]	25 °C	3.53×10^7	2.33×10^7	7.65×10^6	1.10×10^7	9.17×10^6	8.53×10^6	2.73×10^7	3.32×10^7
	37 °C	2.77×10^7	2.32×10^7	8.43×10^6	8.75×10^6	1.02×10^7	1.00×10^7	3.63×10^7	3.07×10^7
	44 °C	1.22×10^6	2.54×10^7	2.99×10^6	9.55×10^6	9.22×10^6	1.00×10^7	9.78×10^6	3.11×10^7
UV exposure[a]	0 minute	1.63×10^7	3.10×10^7	2.80×10^7	4.23×10^7	2.77×10^7	3.03×10^7	1.87×10^7	1.77×10^7
	20 minutes	1.27×10^5	1.77×10^6	1.89×10^5	3.77×10^6	2.57×10^5	1.70×10^4	5.00×10^4	2.00×10^4
	40 minutes	3.23×10^3	1.06×10^6	1.12×10^4	1.00×10^6	2.57×10^3	3.77×10^3	2.11×10^3	2.33×10^3
	60 minutes	1.33×10^2	2.00×10^5	2.43×10^2	1.77×10^5	1.33×10^2	2.10×10^2	3.33×10^1	1.90×10^2
In vivo[b]	0 hour	1.00×10^7	1.00×10^7	1.00×10^7	1.00×10^7	1.00×10^7	1.00×10^7	1.00×10^7	1.00×10^7
	1 hours	1.79×10^1	2.00×10^6	2.50×10^6	1.83×10^6	0	2.53×10^6	1.33×10^5	7.64×10^5
	24 hours	0	1.20×10^6	2.33×10^4	2.50×10^6	0	3.27×10^5	8.10×10^4	1.24×10^5
	48 hours	2.50×10^2	1.64×10^5	1.08×10^4	1.65×10^6	0	3.58×10^5	3.34×10^3	1.36×10^5
	72 hours	0	1.23×10^5	6.25×10^3	2.49×10^6	0	5.75×10^3	7.50×10^2	7.75×10^4
	96 hours	0	5.50×10^4	2.50×10^2	9.74×10^5	0	1.25×10^3	5.00×10^2	7.34×10^4
Storage[c]	Liquid 4 °C	6,52	20.33	3,24	2.29	7.96	8.17	3.41	5.99
	Liquid 25 °C	0	9.35	0.02	2.61	0	0	0.08	3.59
	Powder 4°C	0.09	390.99	13.28	174.11	151.45	163.95	185.32	67.9

	Powder 25°C	0	16.05	9.49	62.23	76.33	19.05	38.73	0.04

	Condition	Distilled Water	5% Skim Milk	0.5% Gelatin	2% Casein	0.5 M Mannitol	0.5 M Sucrose	0.1 M Trehalose	5% Glutamic Acid
Freeze Drying treatment[d]		95.92	93.43	99.99	98.84	100	99.98	99.99	90.07
Leaf Discs Assay[e]	24 hours	52.63	9.21	ND	ND	ND	71.05	ND	21.05
	48 hours	84.27	96.74	ND	ND	ND	9.46	ND	82.18
Kiwi plant Assay[b]	6 hours	0	0	ND	ND	ND	0	ND	0
	24 hours	0	5.55×10^4	ND	ND	ND	2.08×10^4	ND	1.79×10^3
	48 hours	0	0	ND	ND	ND	3.14×10^6	ND	4.10×10^4
Enrichment and detection of phage[f]	6 hours	0	66.6	ND	ND	ND	66.6	ND	33.3
	24 hours	100	66.6	ND	ND	ND	100	ND	66.6
	48 hours	0	100	ND	ND	ND	100	ND	100

[a] Phage titer (PFU/mL)
[b] Phage titer (PFU/g)
[c] Phages titer was normalized as a percentage of the initial titer.
[d] Percentage reduction relative to the phage titer before treatment.
[e] The efficacy percentage of the phage mixture with excipients was calculated relative to the bacterial load of the control experiment without phage at 24 and 48 hours post-inoculation.
[f] Detection of phages after enrichment using Psa.
ND: Not determined.
